# Roles of Chemokines and Chemokine Receptors in Obesity-Associated Insulin Resistance and Nonalcoholic Fatty Liver Disease

**DOI:** 10.3390/biom5031563

**Published:** 2015-07-21

**Authors:** Liang Xu, Hironori Kitade, Yinhua Ni, Tsuguhito Ota

**Affiliations:** Department of Cell Metabolism and Nutrition, Brain/Liver Interface Medicine Research Center, Kanazawa University, Kanazawa 920-8640, Japan; E-Mails: liangxu1023@gmail.com (L.X.); hiro.kitacchi@gmail.com (H.K.); shali0145@gmail.com (Y.N.)

**Keywords:** adipose tissue macrophage, chemokines, inflammation, obesity, insulin resistance, nonalcoholic fatty liver disease, macrophage polarization, Kupffer cells

## Abstract

Abundant evidence has demonstrated that obesity is a state of low-grade chronic inflammation that triggers the release of lipids, aberrant adipokines, pro-inflammatory cytokines, and several chemokines from adipose tissue. This low-grade inflammation underlies the development of insulin resistance and associated metabolic comorbidities such as type 2 diabetes mellitus (T2DM) and nonalcoholic fatty liver disease (NAFLD). During this development, adipose tissue macrophages accumulate through chemokine (C-C motif) receptor 2 and the ligand for this receptor, monocyte chemoattractant protein-1 (MCP-1), is considered to be pivotal for the development of insulin resistance. To date, the chemokine system is known to be comprised of approximately 40 chemokines and 20 chemokine receptors that belong to the seven-transmembrane G protein-coupled receptor family and, as a result, chemokines appear to exhibit a high degree of functional redundancy. Over the past two decades, the physiological and pathological properties of many of these chemokines and their receptors have been elucidated. The present review highlights chemokines and chemokine receptors as key contributing factors that link obesity to insulin resistance, T2DM, and NAFLD.

## 1. Introduction

Obesity is a state of chronic low-grade systemic inflammation that contributes to the development of metabolic diseases such as type 2 diabetes mellitus (T2DM), nonalcoholic fatty liver disease (NAFLD), cardiovascular disease, and several types of cancer [1,2,3,4]. Insulin resistance is known to be an important factor underlying the pathogenesis of these metabolic diseases, but it is also well recognized that adipose tissue acts as an endocrine organ that secretes hormones, or cytokines known as adipokines, such as adiponectin and leptin [5]. Dysregulation of the expression of adipokines caused by adipocyte hypertrophy and dysfunction has been linked to chronic inflammation and insulin resistance through altered immune system function. Obesity-associated systemic inflammation is also characterized by increased circulating concentrations of pro-inflammatory cytokines and chemokines as well as the activation of several kinases that regulate inflammation, including c-Jun NH_2_ terminal kinase (JNK), IκB-kinase β (IKKβ)/nuclear factor κB (NF-κB), and mammalian target of rapamycin (mTOR)/S6 kinase (SK6), which interfere with insulin action in adipocytes and hepatocytes [4].

Advances in obesity research have led to the recognition that obesity-induced inflammation is primarily mediated by immune cells, such as macrophages and T-lymphocytes, in metabolic tissues. In particular, a significant advance in the understanding of obesity-associated inflammation and insulin resistance has been the recognition of the critical role that adipose tissue macrophages (ATMs) play in the body. ATMs are a prominent source of pro-inflammatory cytokines, such as tumor necrosis factor (TNF)-α and interleukin (IL)-6, that can block insulin action in the liver, adipose tissue, and skeletal muscle via autocrine and/or paracrine signaling. Subsequently, this can cause systemic insulin resistance through endocrine signals, which represents a potential link between inflammation and insulin resistance [6,7].

Obesity-associated systemic inflammation in both humans and rodents is characterized by the infiltration of macrophages into adipose tissue and the liver in conjunction with an increase in body weight [8,9,10]. Moreover, both of these factors are positively correlated with insulin resistance. Importantly, macrophages can be classified as M1, or “classically activated” pro-inflammatory macrophages, and M2, or “alternatively activated” non-inflammatory macrophages [11,12]. M2 macrophages predominate in a lean state and help to sustain insulin sensitivity via the secretion of anti-inflammatory cytokines such as IL-4 and IL-10 while M1 macrophages lead to insulin resistance through the secretion of pro-inflammatory cytokines such as tumor necrosis factor-α (TNF-α), IL-6 and IL-1β which, in turn, leads to a pro-inflammatory environment in white adipose tissue (WAT) and the liver [10,12]. Thus, the polarization of M1 and M2 macrophages is closely associated with the development of insulin resistance.

Chemokines are a family of cytokines that induce leukocyte chemotaxis and are deeply involved in the development of allergic diseases and autoimmune diseases. To date, more than 40 chemokines that exhibit various physiological and pathological properties have been discovered [13,14,15]. Previous studies have shown that preadipocytes and adipocytes secrete chemokines, which have been termed monocyte chemoattractant proteins (MCP)-1 (also known as CCL2) [16,17]. MCP-1 causes the infiltration of bone marrow-derived macrophages into obese adipose tissue via binding to the CCR2 receptor and is involved in the development of insulin resistance [16,17]. The present review summarizes the various roles of chemokines and chemokine receptors (the chemokine system) during tissue inflammation with a special focus on the latest findings regarding chemokine systems that represent a link between inflammation in adipose tissue and the liver and the pathologies of insulin resistance, T2DM, and NAFLD.

## 2. Immune Cell Activity during Obesity-Induced Inflammation and Insulin Resistance

### 2.1. Macrophages as a Major Player in Obesity-Induced Insulin Resistance

Overnutrition leads to adipose expansion with hypertrophic adipocytes that secrete various pro-inflammatory cytokines such as TNF-α, IL-1β, and IL-6. These cytokines can downregulate the insulin sensitivity of insulin-sensitive organs, including the liver and skeletal muscle, via the activation of pro-inflammatory signaling and the inhibition of insulin receptor signaling [18]. Hypertrophic adipocytes also secrete MCP-1, which promotes the infiltration of monocytes into adipose tissue where they differentiate into macrophages and promote inflammation via the secretion of pro-inflammatory cytokines.

Hepatic insulin resistance during obesity has been linked to inflammation due to the activation of pro-inflammatory signaling and the accumulation of intracellular lipids. For example, pro-inflammatory pathways in Kupffer cells, which are the resident hepatic macrophages, are activated during obesity and contribute to the production of inflammatory mediators that promote insulin resistance and fatty liver disease. In fact, the removal of Kupffer cells from the liver results in an attenuation of hepatic insulin resistance during high-fat feeding [19] as well as an increase in the number of macrophages and decreased glucose uptake in the skeletal muscle of obese mice [20]. Thus, immune cells that can infiltrate insulin-sensitive tissues such as fat, the liver, and muscle are associated with tissue inflammation and contribute to insulin resistance during obesity.

### 2.2. Macrophage Plasticity and Polarization

Tissue macrophages are phenotypically heterogeneous and can be characterized according to their activation/polarization state as M1, or “classically activated” pro-inflammatory macrophages, and M2, or “alternatively activated” non-inflammatory macrophages [11,12]. M1 macrophages secrete various pro-inflammatory cytokines, such as TNF-α and IL-6, which induce insulin resistance via the IKKβ- and JNK-mediated inhibitory serine phosphorylation of insulin receptor substrate (IRS) proteins [21]. M1-polarized macrophages express CD11c and account for the majority of the increase in ATMs observed in obese adipose tissue [22,23]. In contrast, M2-polarized macrophages secrete anti-inflammatory cytokines, such as IL-10 and IL-1 receptor antagonist (IL-1Ra) [24]. M2 macrophages do not express CD11c but do express prototypical M2 markers that include IL-10, Ym1 (chitinase 3), arginase, and macrophage galactose-type lectin 1 (MGL 1) [12]. In mice, IL-4 treatment induces the M2-associated activation of ATMs, which in turn attenuates high fat diet (HFD)-induced insulin resistance [25]. Similarly, IL-10 secreted by M2 macrophages enhances insulin signaling in the liver and skeletal muscle and protects against obesity-induced insulin resistance [26]. Thus, M1 macrophages play a critical role in the development of inflammation and insulin resistance whereas the resident M2 macrophages function during anti-inflammatory responses and tissue homeostasis.

### 2.3. The Role of Other Immune Cells during Insulin Resistance

Recent studies have demonstrated that other immune cells, including CD4^+^ and CD8^+^ T-cells, natural killer T- (NKT) cells, B-cells, eosinophils, neutrophils, and mast cells, can be found in adipose tissue and that they likely play important roles during inflammation and insulin resistance. Nishimura *et al.* [27] reported that the number of CD8^+^ T-cells is increased in obese adipose tissue and that CD8^+^ infiltration precedes and promotes HFD-induced ATM accumulation while CD8-deficient mice exhibit improved insulin sensitivity. In contrast, CD4^+^ T-cells and regulatory T- (Treg) cells are resident during a lean state and induce the alternative activation of macrophages, which improves insulin sensitivity [28,29].

B-cells also accumulate in the obese adipose tissue of HFD-fed mice, where they promote the activation of T-cells, which in turn potentiate M1 macrophage polarization and insulin resistance [30]. B-cell-deficient mice and mice treated with a B-cell-depleting CD20 antibody in conjunction with a HFD diet exhibit an amelioration of systemic inflammation, decreased numbers of inflammatory cytokines, reduced adipose tissue inflammation, and improved insulin resistance compared with obese wild-type (WT) mice [30,31]. These changes were associated with an increase in the number of anti-inflammatory Treg cells.

Eosinophils also play a role during M2 activation by secreting IL-4 in lean adipose tissue and contributing to insulin sensitivity [32]. Similar to eosinophils, NKT cells also reside in lean adipose tissue and sustain M2 macrophages through the activation of IL-4/STAT6 signaling [25]. Thus, the activation of eosinophils or NKT cells results in improved glucose tolerance and insulin resistance. Additionally, recent studies have shown that neutrophils induce adipose tissue inflammation and insulin resistance in HFD-induced obese mice by secreting elastase [33] and that mast cells infiltrate adipose tissue during obesity and not only contribute to an increase of pro-inflammatory cytokines and chemokines but also the promotion of tissue inflammation and insulin resistance [34]. On the other hand, the depletion of neutrophil elastase or mast cells in HFD-induced obese mice results in improved glucose tolerance and increased insulin sensitivity [33,35]. Therefore, an increase in the levels of neutrophils or mast cells could contribute to inflammation and insulin resistance.

## 3. Regulation of Insulin Resistance and Inflammation in Adipose Tissue by the Chemokine System

### 3.1. Classification of Chemokines and Chemokine Receptors

Chemokines are a family of low-molecular-weight cytokines that play central roles in the trafficking of leukocytes to lesions and areas of inflammation as well as leukocyte activation. Chemokines were first described as cytokines that are chemotactic for neutrophils and monocytes and that are involved in the development of allergies and autoimmune diseases. A number of studies have investigated the roles of chemokines in acute neutrophil-predominant inflammation and chronic monocyte- and lymphocyte-predominant inflammation [36,37]. On the basis of their molecular structure, which includes two N-terminal cysteine residues, chemokines are classified into the following four subfamilies: CXC, CC, C, and CX3C, where X is any amino acid residue (Table 1). CXC chemokines exhibit mainly neutrophils chemotaxis and are known for their involvement in acute inflammation, whereas most of the CC chemokines act on monocytes, T-cells, eosinophils, and basophils to mediate chronic inflammation and allergies [13].

**Table 1:** Chemokines and chemokine receptors.

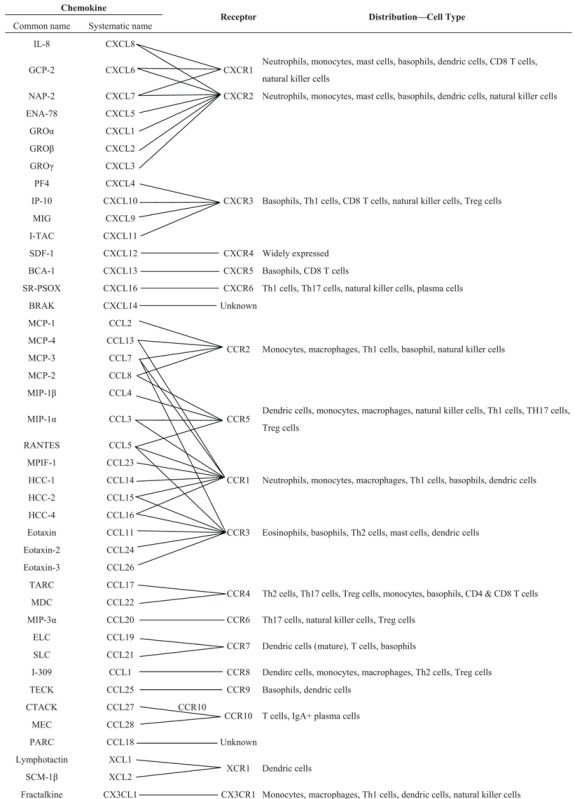

Chemokine receptors belong to the seven-transmembrane G-protein-coupled receptor superfamily and consist of single polypeptide chains with three extracellular loops and three intracellular loops [13,14,15]. To date, 19 chemokine receptors have been identified: eleven CC chemokine receptors (CCR1-CCR11), six CXC chemokine receptors (CXCR1-CXCR6), one C chemokine receptor (XCR1), and one CX3C chemokine receptor (CX3CR1; Table 1). In general, the chemokine system functions to maintain homeostasis or induce inflammation. However, there is the inherent redundancy of the chemokine system; some chemokines have a specific receptor but most chemokine ligands share the receptor (Table 1). For example, four chemokine ligands—CCL2 (also known as MCP-1; (MCP-1/CCL2), MCP-2/CCL8, MCP-3/CCL7, and MCP-4/CCL13—bind to the CCR2 receptor, whereas CX3CL1 (also known as fractalkine) specifically binds to CX3CR1 (Table 1). However, although multiple ligands can bind to a single receptor, they exert different effects because their binding affinities differ [13,15]. Furthermore, because chemokines are expressed, distributed, and regulated in different cells and tissues, they may play different roles in a variety of conditions or diseases.

### 3.2. Role of the MCP-1-CCR2 System in ATM Recruitment

The interaction of MCP-1 with its receptor, CCR2, is considered pivotal for the recruitment of ATMs and the development of obesity-induced insulin resistance. Several groups have reported that CCR2- and MCP-1-deficient mice exhibit decreased ATM content, attenuated inflammation in adipose tissue, and protection against HFD-induced insulin resistance [16,17]. Conversely, adipose tissue-specific overexpression of MCP-1 in mice is sufficient to increase the number of ATMs as well as insulin resistance [38]. Additionally, mice treated with a pharmacological antagonist of CCR2 show a decrease in ATM content and improved insulin sensitivity without a decrease in body weight [39]. Therefore, MCP-1-CCR2 signaling plays a central role during the promotion of ATM recruitment and insulin resistance.

However, more recent studies have reported conflicting results and suggest that a greater degree of complexity is involved in this process than indicated by earlier reports. For example, MCP-1-deficient mice do not exhibit reduced macrophage accumulation or improved metabolic function, which suggests that MCP-1 is not critical for obesity-induced ATM recruitment or systemic insulin resistance [40,41]. Furthermore, although *Ccr2*^−/−^ mice fed a HFD have fewer macrophages in their WAT than WT mice, the CCR2 deficiency does not normalize ATM content or insulin resistance to the levels of lean animals [16]. These findings suggest that not all types of ATM recruitment or the associated insulin resistance are regulated by MCP-1-CCR2 signaling.

The complexity and redundancy of chemokine signaling may be relevant to the recruitment of macrophages and the induction of inflammation and insulin resistance. In fact, chemokine systems other than the MCP-1-CCR2 signaling pathway have been implicated in the infiltration of ATMs in obese mice. ENA-78/CXCL5, which is secreted by macrophages, has been linked to inflammation in adipose tissue and insulin resistance [42]. There is an increase in the serum levels of ENA-78/CXCL5 in obese mice and humans, and ENA-78/CXCL5 inhibits insulin signaling through its receptor CXCR2. Additionally, mice lacking CXCR2 show resistance to the onset of obesity-induced disorders of glucose metabolism, and a recent study found that SDF-1/CXCL12, which is secreted from hypertrophic adipocytes, recruits macrophages via CXCR4, which is the receptor of SDF-1/CXCL12, during obesity [43]. Circulating levels of SDF-1/CXCL12 are also increased in HFD-induced obese mice and it has been shown that SDF-1/CXCL12-CXCR4 signaling induces M1 macrophage accumulation. The blockade of SDF-1/CXCL12-CXCR4 signaling results in the reduced recruitment of macrophages and the diminished secretion of pro-inflammatory cytokines in WAT as well as improved insulin resistance. However, additional unidentified chemokines and chemokine receptor pathways that may play significant roles in ATM recruitment and insulin sensitivity remain to be fully characterized.

**Figure 1:**
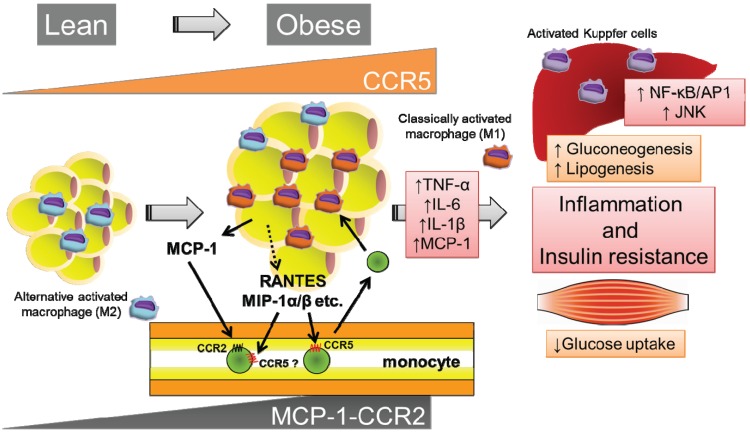
CCR5 promotes obesity-induced inflammation and insulin resistance. In a lean state, M2 macrophages are the primary resident macrophages and maintain insulin sensitivity. On the other hand, over nutrition and/or a lack of exercise can cause adipocyte hypertrophy, which initiates the secretion of MCP-1 and leads to the recruitment of circulating monocytes to adipose tissues. Subsequently, CCR2^+^ macrophages accumulate and presumably maintain inflammation as M1 macrophages in obese adipose tissue. Similarly, CCR5^+^ adipose tissue macrophages (ATMs) infiltrate and promote the inflammatory response. Once these ATMs are present and active, they can maintain a vicious cycle involving ATM recruitment and the production of inflammatory cytokines such as TNF-α, IL-6, and IL-1β in conjunction with adipocytes and other infiltrated immune cells. These secreted pro-inflammatory cytokines subsequently cause insulin resistance in adipose tissue, the liver, and skeletal muscle via the activation of several kinases, including JNK and NF-κB/AP1. Therefore, CCR5, independently from and/or cooperatively with CCR2, plays a pivotal role in the induction and maintenance of obesity-induced inflammation and insulin resistance.

### 3.3. CCR5 Regulates Insulin Resistance and the Recruitment and Polarization of ATMs

A critical role for CCR5, which is a different CC chemokine receptor, during the regulation of the inflammatory response to obesity in adipose tissue and the development of systemic insulin resistance (Figure 1) has recently been identified and characterized by our research group [44]. This study produced several important observations. First, under obese conditions, the expressions of CCR5 and its ligands are significantly increased and equal to that of CCR2 and its ligands in the WAT of genetically (*ob*/*ob*) and HFD-induced obese mice, particularly in the macrophage fraction. Second, the number of CCR5^+^ ATMs robustly increases in response to a HFD. Third, *Ccr5*^−/−^ mice are protected from impairments in HFD-induced glucose homeostasis and hepatic steatosis. Importantly, the infiltration of macrophages into adipose tissue and crown-like structure formations in obese adipose tissue are markedly reduced in *Ccr5*^−/−^ mice despite there being no significant differences in adipocyte size between *Ccr5*^−/−^ and WT mice. Furthermore, chimeric mice lacking CCR5 only in the myeloid cells are protected from HFD-induced hyperinsulinemia and glucose intolerance through reductions in ATM accumulation. Finally, CCR5 deficiency causes an M2-dominant phenotypic shift in ATMs. Taken together, these findings demonstrate that CCR5 plays a critical role during the inflammatory response to obesity in adipose tissue via the regulation of macrophage recruitment and M1/M2 macrophage polarization. In light of these data, CCR5 may be a promising therapeutic target for patients with insulin resistance and T2DM. However, several questions have yet to be answered, including what distinct roles are played by CCR5 of the 40 chemokines in metabolic disease, and are there interactions between CCR2 signaling and CCR5 signaling? Further work is required to gain a systematic understanding of how CCR5 and MCP-1-CCR2 as well as other chemokine systems, connect obesity, inflammation, and insulin resistance.

## 4. Role of the Chemokine System during the Progression from NAFLD to NASH

### 4.1. Progression of NAFLD to NASH

**Figure 2:**
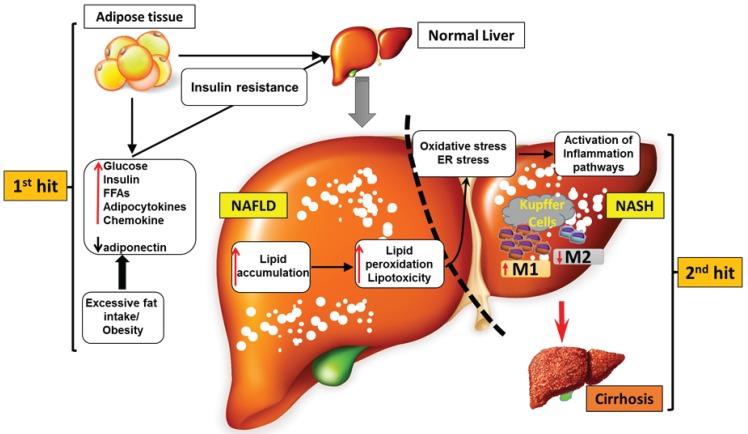
Pathomechanisms during the progression to NASH. The development of NASH is initiated by several different risk factors including a high-fat diet, physical inactivity, and genetic predispositions that often lead to obesity and insulin resistance. Exaggerated fat intake and obesity lead to hyperglycemia, hyperlipidemia, and the over expressions of adipocytokines and chemokines and further contribute to insulin resistance in adipose tissue and the liver. Insulin resistance results in hepatic triglyceride (TG) synthesis and the increased delivery of free fatty acids (FFAs) to the liver. Additionally, hepatic steatosis acts as a “first hit” that is followed by a “second hit” in which inflammatory mediators can cause NASH and even cirrhosis. An enhanced storage of TG provokes a series of harmful consequences related to hepatocytes, such as uncontrolled lipid peroxidation, oxidative stress, and endoplasmic reticulum (ER) stress, which can activate hepatic inflammatory pathways. In particular, the recruitment of macrophage/Kupffer cells and an M1-dominant phenotypic shift in macrophages in the liver play a key role in the pathological progression of NASH.

NAFLD is characterized by excessive fat accumulation in the form of triglycerides (TG) in the liver and has become the most common cause of chronic liver disease in wealthy countries. NAFLD encompasses a spectrum of diseases ranging from simple steatosis (>5% of liver weight) to nonalcoholic steatohepatitis (NASH), which may develop into hepatic fibrosis, cirrhosis, or hepatic carcinoma (Figure 2) [45]. Additionally, NAFLD and NASH are generally considered to have strong associations with insulin resistance, obesity, T2DM, dyslipidemia, and other metabolic syndromes. Although the pathogenesis of NAFLD is varied and complex, the traditional two-hit theory of its development is widely accepted [46]. The first hit refers to the accumulation of TG in hepatocytes that results from an imbalanced hepatocellular lipid metabolism. This is followed by the second hit, which is an increase in inflammatory mediators such as cytokines, chemokines, and adipocytokines that causes hepatocellular injury, inflammation, and fibrosis (Figure 2). Recently, however, this classic pathophysiological theory has been challenged by new findings regarding the interactions among insulin resistance, adipokines, adipose tissue inflammation, and other less-recognized pathogenetic variables. Thus, a new model in which the occurrence of multiple parallel hits is likely responsible for the development of NAFLD has been proposed [47].

NASH involves hepatocellular steatosis in conjunction with liver inflammation and hepatocyte lesions. A variety of hepatocellular factors—including hepatocytes, hepatic macrophages, and hepatic stellate cells—contribute to the various forms of NASH and, of these cells, hepatic macrophages consisting of resident Kupffer cells and recruited bone marrow-derived macrophages represent the major producer of inflammatory mediators such as TNFα, IL-1β, and reactive oxygen species (ROS). These inflammatory mediators further stimulate the induction of hepatocyte steatosis and fibrosis by hepatocytes and hepatic stellate cells, respectively [48].

### 4.2. CCRs in NAFLD and NASH

Of the many chemokines and chemokine receptors, the hepatic expression of MCP-1-CCR2 is upregulated and plays an important role during the induction of hepatic inflammation and insulin resistance. The expression of MCP-1 in hepatocytes is increased in animals fed a HFD and leads to the hepatic recruitment of CCR2^+^ myeloid cells that promote hepatic steatosis [10]. The MCP-1-CCR2 pathway is also upregulated in the livers of animals with diet-induced NASH [48] and MCP-1-CCR2 signaling involves hepatic fibrosis through the promotion of the migration of hepatic stellate cells [49]. In contrast, it has been shown that the inactivation of CCR2 inhibits NASH in the liver [48] and that the genetic deletion of MCP-1 and CCR2 attenuates obesity and improves insulin resistance and hepatic steatosis [16,17]. Collectively, MCP-1-CCR2 signaling is central to the potential progression of simple steatosis to NASH.

RANTES/CCL5 is primarily involved in the migration of T-cells, monocytes, neutrophils, and dendritic cells through binding to its cognate transmembrane receptors, CCR1, CCR3, and CCR5. The CCR5 receptor has been identified on isolated hepatic stellate cells, which suggests that these hepatic cells are both the target and source of RANTES/CCL5 [50]. The expression of CCR5 is upregulated in diet-induced obese mice and obese patients [44,51] and the inactivation of CCR5 protects mice from insulin resistance and hepatic fatty infiltration and allows a shift in the polarization of macrophages toward the M2 phenotype [44]. Hepatocytes are the major source of serum and hepatic RANTES/CCL5 in NAFLD and NASH and this is mediated by the deposition of TG in hepatocytes. In fact, RANTES/CCL5 is also involved in the progression of hepatic fibrosis in mice via the triggering of CCR1 and CCR5 [50]. Taken together, these findings indicate that the RANTES/CCL5-CCR5 axis is a promising therapeutic target for the reduction of NAFLD and NASH.

In addition to MCP-1 and RANTES, other chemokines such as IL-8/CXCL8, MIG/CXCL9, and IP-10/CXCL10 are also involved in the mediation of NAFLD and NASH [52]. CXCL8 (also known as IL-8) is a CXC chemokine subfamily produced by several cell types, including inflammatory and endothelial cells. The function of IL-8/CXCL8 is to orchestrate neutrophil recruitment within inflamed tissues. Serum levels of IL-8/CXCL8 are independently associated with NASH and significantly higher in subjects with NASH compared to subjects with hepatosteatosis or healthy controls [53]. On the other hand, MIG/CXCL9 and IP-10/CXCL10 share the common receptor CXCR3, which is highly expressed in activated T-cells, memory T-cells, and natural killer cells [52]. Moreover, high levels of MIG/CXCL9 are found in the livers of patients with NASH, which suggests that the MIG/CXCL9-CXCR3 axis is a potential target for the treatment of liver fibrosis in both humans and animals [54].

### 4.3. The Polarization of Liver M1/M2 Macrophage Regulates Insulin Resistance and NASH

NAFLD is associated with chronic inflammation of the liver and adipose tissue via the infiltration of immune cells such as dendritic cells, macrophages, and T-lymphocytes [18]. Consequently, ATMs play a key role in insulin resistance in adipose tissue and the release of excess free fatty acids (FFAs) into the circulation or portal vein and, thus, the deposition of ectopic fat in the liver. Kupffer cells are the largest resident population of macrophages in the liver and can differentiate into M1 and M2 macrophages [55]. Clinical findings and experimental data have demonstrated that the activation of Kupffer cells is a central event in the initiation of liver injury. As a result, the exacerbated release of M1 macrophage/Kupffer cell-derived mediators contributes to the pathogenesis of several liver lesions, including hepatocyte steatosis and apoptosis, the recruitment of inflammatory cells, and the activation of fibrogenesis [55,56]. TNF-α and chemokines such as MCP-1 and RANTES/CCL5 that are produced by M1-activated macrophages induce hepatic cholesterologenesis and increase TG production, which results in the discordant regulation of lipid metabolism and homeostasis [57]. Moreover, the alternatively activated M2-polarized Kupffer cells represent another critical pathway for the resolution of inflammatory responses in subjects with NAFLD. M2 Kupffer cells promote the caspase-3-dependent apoptosis of classically activated M1 Kupffer cells and provide a protective mechanism against NAFLD [56]. Thus, the ratio of M1/M2 is increased during the progression of NAFLD and the polarization of cells into M2 Kupffer cells might protect against fatty liver disease.

### 4.4. Chemokines as a Therapeutic Target for the Treatment of NASH: The Emerging Role of β-Cryptoxanthin

As reviewed and discussed thus far, the recruitment of macrophages, which is in some manner regulated by the chemokine system, is a pivotal factor in obesity-associated insulin resistance and metabolic diseases such as NAFLD and NASH. However, several promising treatments targeting the hepatic activation and polarization of macrophages in patients with NASH are being developed. β-Cryptoxanthin is a xanthophyll carotenoid that is relatively abundant in human plasma. Epidemiological studies have demonstrated that high levels of serum β-cryptoxanthin are associated with diminished insulin resistance and alcoholic liver injury in non-diabetic subjects [58,59]. Additionally, *in vivo* and *in vitro* studies have shown that β-cryptoxanthin exerts anti-inflammatory effects that primarily operate via the modulation of the innate immune response induced by macrophages [60]. Of note, the gene expressions of chemokines, including MCP-1, IP-10/CXCL10, and MIP-1α/CCL3, and pro-inflammatory cytokines, including TNFα, IL-1β, and IL-6, in the liver and adipose tissue are markedly decreased by β-cryptoxanthin [61]. These findings indicate that M1-polarized macrophages *per se* or chemokines may represent a therapeutic target for the treatment of insulin resistance, metabolic syndrome, and NAFLD.

**Figure 3:**
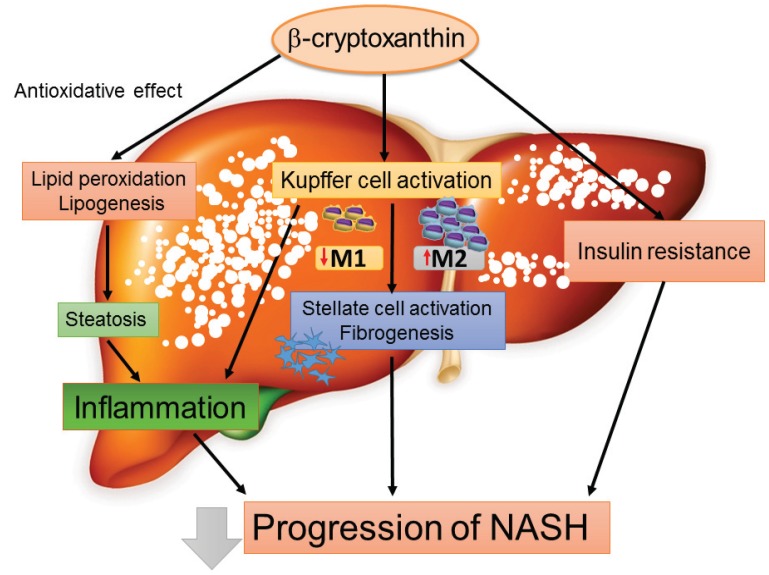
Beneficial effects of β-cryptoxanthin on the progression of NASH. β-Cryptoxanthin, an antioxidant carotenoid, inhibits the progression of NASH by attenuating lipid accumulation, lipid peroxidation, and insulin resistance. Furthermore, β-cryptoxanthin decreases numbers of M1 macrophages while increasing those of M2 macrophages, which results in an M2-dominant shift in Kupffer cells and leads to an attenuation of lipid-induced insulin resistance, inflammation, and fibrosis in NASH.

Importantly, our research group recently demonstrated that β-cryptoxanthin prevents and reverses insulin resistance and NASH in mice by suppressing excessive lipid accumulation and peroxidation [62,63]. A schematic representation of the beneficial effects of β-cryptoxanthin on the progression of NASH is provided in Figure 3. β-Cryptoxanthin inhibits the progression of NASH by attenuating lipid accumulation, lipid peroxidation, and insulin resistance as well as reducing the accumulation of T-cells and regulating the M1/M2 status of Kupffer cells in the liver, which occurs at least partly through a downregulation of the MCP-1-CCR2 and RANTES/CCL5-CCR5 pathways [62]. More specifically, β-cryptoxanthin decreases M1 macrophages while increasing M2 macrophages, which results in an M2-dominant shift in Kupffer cells and leads to an attenuation of lipid-induced insulin resistance and inflammation in NASH [63]. Otherwise, the beneficial effects of β-cryptoxanthin are due to a decrease in the hepatic recruitment of T-cells and macrophages and an M2-dominant shift in macrophages/Kupffer cells. Thus, β-cryptoxanthin may be a promising treatment for NASH via reductions in the chemokine-mediated recruitment of immune cells and an M2-dominant shift in macrophages/Kupffer cells.

## 5. Conclusions and Perspectives

It has become increasingly evident that chemokines and chemokine receptors play an important role in obesity-induced insulin resistance and comorbid diseases such as T2DM, cardiovascular disease, and NAFLD and NASH. MCP-1-CCR2 and other chemokine systems such as CCR5 may be integrally involved in tissue- and organ-level inflammation caused by interactions among adipose tissue, the liver, and macrophages as well as the subsequent development of systemic insulin resistance and metabolic disorders.

However, many questions remain to be answered, including the manner in which chemokine expression is regulated during obesity, how chemokines regulate the polarization of macrophages, and the specific roles of the over 40 chemokines in metabolic diseases. Additionally, the involvement of chemokines and their receptors in the pathogenesis of NAFLD is only partially understood. Taken together, all of the evidence supporting the interrelationships among chemokines and NAFLD provides important information for the treatment of NAFLD and NASH. Further research into the relationships of chemokines with the molecular bases of metabolic inflammation and the pathogenesis of insulin resistance is expected to contribute to the development of novel drugs that could control inflammation- or immune-mediated disorders such as metabolic syndrome, T2DM, and NAFLD.
